# Evaluation of multiple institutions’ models for knowledge-based planning of volumetric modulated arc therapy (VMAT) for prostate cancer

**DOI:** 10.1186/s13014-018-0994-1

**Published:** 2018-03-20

**Authors:** Yoshihiro Ueda, Jun-ichi Fukunaga, Tatsuya Kamima, Yumiko Adachi, Kiyoshi Nakamatsu, Hajime Monzen

**Affiliations:** 1 Department of Radiation Oncology, Osaka International Cancer Institute, 3-1-69 Otemae, Chuo-ku, Osaka, 537-8567 Japan; 2 Divisin of Radiology, Department of Medical Technology, Kyushu University Hospital, Maidashi, Higashi-ku, Fukuoka, 812-8582 Japan; 3 Department of Radiation Oncology, Cancer Institute Hospital, Japanese Foundation for Cancer Research, 3-8-31 Ariake, Koto-ku, Tokyo, 135-8550 Japan; 4 Department of Radiology, Seirei Hamamatsu General Hospital, 2-12-12 Sumiyoshi, Naka Ward, Hamamatsu, Shizuoka 430-8558 Japan; 5 Department of Radiation Oncology, Faculty of Medicine, Kindai University, 377-2 Ohno-higashi, Osakasayama, Osaka, 589-8511 Japan; 6 Department of Medical Physics, Graduate School of Medical Sciences, Kindai University, 377-2 Ohno-higashi, Osakasayama, Osaka, 589-8511 Japan

**Keywords:** Knowledge-based planning, Inverse planning, Prostate cancer, Quality assurance for planning, RapidPlan

## Abstract

**Background:**

The aim of this study was to evaluate the performance of a commercial knowledge-based planning system, in volumetric modulated arc therapy for prostate cancer at multiple radiation therapy departments.

**Methods:**

In each institute, > 20 cases were assessed. For the knowledge-based planning, the estimated dose (ED) based on geometric and dosimetric information of plans was generated in the model. Lower and upper limits of estimated dose were saved as dose volume histograms for each organ at risk. To verify whether the models performed correctly, KBP was compared with manual optimization planning in two cases. The relationships between the EDs in the models and the ratio of the OAR volumes overlapping volume with PTV to the whole organ volume (V_overlap_/V_whole_) were investigated.

**Results:**

There were no significant dosimetric differences in OARs and PTV between manual optimization planning and knowledge-based planning. In knowledge-based planning, the difference in the volume ratio of receiving 90% and 50% of the prescribed dose (V90 and V50) between institutes were more than 5.0% and 10.0%, respectively. The calculated doses with knowledge-based planning were between the upper and lower limits of ED or slightly under the lower limit of ED. The relationships between the lower limit of ED and V_overlap_/V_whole_ were different among the models. In the V90 and V50 for the rectum, the maximum differences between the lower limit of ED among institutes were 8.2% and 53.5% when V_overlap_/V_whole_ for the rectum was 10%. In the V90 and V50 for the bladder, the maximum differences of the lower limit of ED among institutes were 15.1% and 33.1% when V_overlap_/V_whole_ for the bladder was 10%.

**Conclusion:**

Organs’ upper and lower limits of ED in the models correlated closely with the V_overlap_/V_whole_. It is important to determine whether the models in KBP match a different institute’s plan design before the models can be shared.

## Background

The plan quality for intensity-modulated radiotherapy (IMRT) and volumetric-modulated arc therapy (VMAT), which are created by inverse planning, depends on the planner’s or institution’s experience and skills [1–3]. Institutional experience substantially influences survival in locally advanced head and neck cancer [4]. Some studies have suggested methods to verify the quality of plans created by inverse planning [5–7].

For quality assurance of an inverse planning algorithm, Moore et al. [5] reported that predicting the dose to an organ at risk (OAR) from the volume of the OAR within the planning target volume (PTV) was useful to reduce variations in planning quality. Recently, a new assistance tool for inverse planning, RapidPlan (Varian Medical Systems, Palo Alto CA, USA), which performs knowledge-based planning (KBP), was developed and released for clinical use. Details of the system have been described in a previous study [8]. Some studies have suggested that the performance of KBP be compared with manually-optimized plans for clinical use. They mentioned that KBP is superior to manual planning in reducing OAR dose [9–12].

The KBP system has the advantage that its model is shared by multiple institutions. Sharing models is considered to be a good method for reducing variability in planning quality among multiple institutions. There has been no report that KBP with the models in multiple institutions was employed for the same CT data. The aim of this study was to evaluate the performance of KBP models in multiple institutions to optimize the model.

## Methods

### Institutes and plan design

In this study, five institutes (A–E) were enrolled. These institutes treated patients with T1–T2c prostate cancer using VMAT. Table 1 shows the definition of gross tumor volume (GTV), margins to define the clinical target volume (CTV) and PTV in each direction. In each institution, the dose constraints are shown in Table 2. The five institutes had different plan designs.

**Table 1:** The definition of gross tumor volume (GTV), margins to define the clinical target volume (CTV) and PTV in each direction

Institute	Gross tumor volume (GTV)	GTV to CTV margin [mm]						CTV to PTV margin [mm]					
		A	P	S	I	L	R	A	P	S	I	L	R
A	Prostate and proximal 20 mm of SV	0	3	3	0	3	3	4	4	4	4	4	4
B	Prostate and proximal 15 mm of SV	0	0	0	0	0	0	8	5	8	8	8	8
C	Prostate and proximal 10 mm of SV	0	0	0	0	0	0	6	4	6	6	6	6
D	Prostate	3	3	3	3	3	3	3	3	3	3	3	3
E	Prostate and the half of SV	0	0	0	0	0	0	8	6	8	8	8	8

*Abbreviations: GTV* the gross tumor volume, *CTV* the clinical target volume, *PTV* the planning target volume, *SV* the seminal vesicle, *A* the anterior direction, *P* the posterior direction, *S* the superior direction, *I* inferior direction, *L* the left direction, *R* the right direction

**Table 2:** Dose constraints for treatment of prostate cancer using volumetric-modulated arc therapy in each institution

Institute	Organ		Target	
	Rectum	Bladder	CTV	PTV
A	Ractal wall	Bladder wall	CTV	PTV
	V78 (Gy) ≤ 0.1 cm^3^	V70 (Gy) ≤ 35%	Dmin ≥100%	D50 = 100%
	V70 (Gy) ≤ 25%	V40 (Gy) ≤ 60%		
	V60 (Gy) ≤ 35%			
	V40 (Gy) ≤ 60%			
B	Ractal wall	Bladder wall	CTV	PTV sub. Rectum
	V78 (Gy) < 1%	V70 (Gy) < 35%		Dmean < 103%
	V70 (Gy) < 20%	V40 (Gy) < 60%		Dmin > 99%
	V60 (Gy) < 30%			Dmax < 110%
	V40 (Gy) < 60%			D95 = 100%
C	Rectum	Bladder	CTV	PTV
	V70 (Gy) ≤ 5%	V80 (Gy) ≤ 5%	D98 ≥ 98%	Dmean = 100%
	V65 (Gy) ≤ 10%	V75 (Gy) ≤ 15%	D2 ≤ 105%	D95 ≥ 95%
	V60 (Gy) ≤ 20%	V70 (Gy) ≤ 25%		V90 ≥ 98%
	V40 (Gy) ≤ 40%	V60 (Gy) ≤ 40%		D2 ≤ 105%
D	Rectum sub. PTV	Bladder sub. PTV	CTV	PTV sub. (Rectum and bladder)
	D50 ≤ 69.7%	D5 ≤ 78.9%	D95 = 100%	68.4% ≤ D5 ≤ 71.1%
	D5 ≤ 78.9%	D50 ≤ 72.4%	88.2% ≤ D5 ≤ 92.1%	65.8% ≤ D50 ≤ 71.1%
			85.5% ≤ D50 ≤ 88.2	64.5% ≤ D95 ≤ 68.4%
			81.6% ≤ D95 ≤ 85.5	
E	Ractal wall	Bladder wall	CTV	PTV
	V78 (Gy) ≤ 1%	V70 (Gy) ≤ 35%		Dmean = 100%
	V70 (Gy) ≤ 20%	V40 (Gy) ≤ 60%		D95 ≥ 95%
	V60 (Gy) ≤ 35%			V90 ≥ 98%
	V40 (Gy) ≤ 60%			Dmax ≤110%

*Abbreviations: CTV* the clinical target volume, *PTV* the planning target volume, *Dmean* mean dose, *Dmin* minimum dose, *Dmax* maximum dose, *V80, V78, V70, V65, V60, and V40* the OAR volume ratio that receives a dose exceeding 80 Gy, 78 Gy, 70 Gy, 65 Gy, 60 Gy, and 40 Gy, *V90* the volume ratio receiving 90% of the prescribed dose, *D95, D50, D5, and D2* the dose received by at least 95%, 50%, 5.0%, and 2.0% of the volume

### The model for KBP and exporting the estimated dose

In each institute, the model for KBP was created using the VMAT plans for clinical use at each institute before April 2017. The number of registered cases in institute A, institute B, institute C, institute D, and institute E were 123, 53, 20, 60, and 100, respectively.

Users performed three main steps to create models for KBP. In the first step, dose volume histogram (DVH) estimation model configuration, > 20 plans that had been used in clinical settings were registered. The next step was the extraction phase. In each OAR of registered plans, dosimetric and geometric information was imported in the model. The last step was the training step, based on the information from the extraction phase. In this step, in each OAR of registered plans new DVH curves were generated. Upper and lower limits of the estimated doses (ED) were obtained. These dose limits were saved in the form of DVH in the model. To attain the ideal dose distribution, the parameters, except line objects shown in Table 3, were set in some institutes.

**Table 3:** Objectives except line objects in models

	Organ					Target			
	Objective type	Volume	Dose	gEUD	Priority	Objective type	Volume	Dose	Priority
A	Rectum					CTV			
	Upper	0	100%		58	Upper	0	104%	100
	Bladder					Lower	100	100%	63
	Upper	0	100%		58	PTV			
						Upper	0	104%	100
						Lower	100	90%	63
B	Rectum					CTV			
	Upper	0	98%		45–50				
	Bladder					PTV			
						Upper	0	102%	120
						Lower	100%	100%	120
C	Rectum					CTV			
	Upper	0	100%		generate				
	Upper gEUD		69.2%	9	generate	PTV			
	Bladder					Upper	0	100%	115
						Lower	100%	93%	generate
						Lower	97%	96%	generate
						Lower	95.50%	98%	generate
						Lower	90%	100%	generate
D	Rectum					CTV			
	Upper	0	78.9%		generate	Upper	0	102.6%	generate
	Bladder					Lower	95%	100%	generate
	Upper	0	81.6%		generate	Lower	100%	97.4	generate
						PTV			
						Upper	0	97.4%	generate
						Lower	0	94.7%	generate
E	Rectum					CTV			
	Upper	0	99.0%		generate	Lower	100.0%	99.0%	generate
	Upper gEUD		77.0%	10.0	generate	PTV			
	Bladder					Upper	0.0%	103.0%	generate
						Lower	100.0%	90.0%	generate
						Lower	97.0%	97.0%	generate

*Abbreviations: CTV* the clinical target volume, *PTV* the planning target volume, *gEUD* generalized equivalent uniform dose

These data were read from an .xml file exported to the website of Model Analytics (https://ModelAnalytics.varian.com). The file also contained basic information on the model, such as original and estimated DVH data and OAR volume, and the ratio of an OAR’s volume overlapping with PTV to the whole organ volume (V_overlap_/V_whole_). To evaluate the performance in reducing the dose to rectum and bladder in each model, the original DVH, and upper and lower limits of ED, were extracted from the file.

### Calculation of dose distributions with manual optimization and KBP

To investigate whether KBP was performed correctly, two sets of CT data and structures of patients at institute B were anonymized and delivered to other institutes. Written informed consent was obtained from all patients, and the Institutional Ethics Committee approved this study (Kindai University review board number: 29–133). The thickness of the CT sections was 2.5 mm and the field of view was 50 cm. The target and OARs were contoured by a physician according to the protocol of institute B. The bladder in one case (case I) had a volume of 83.8 cm^3^, in another case (case II), bladder volume of 181.8 cm^3^. V_overlap_/V_whole_ of the rectum and bladder were 9.8% and 11.1% in case I and 5.9% and 5.9% in case II, respectively.

At each institute, the planners who participated in this study had experience with inverse planning for IMRT or VMAT with the Eclipse (Varian Medical Systems, Palo Alto CA, USA) treatment planning system (TPS). They attended a special lecture (RapidPlan Clinical Advisory Board) on Rapidplan held by the manufacturer in Tokyo in June 2017. In KBP using Rapidplan, single optimization was performed. Next, in the manual optimization planning, the optimization was repeated until it achieved the institutional ideal dose distribution. In manual optimization, the generalized equivalent uniform dose (gEUD) was not used in all institutes. In KBP and manual optimization planning, the same calculation parameters and beam parameters were used. The photon optimization was used with 2.5 mm grid size. The calculation algorithm was Anisotropic Analytical Algorithm ver. 13.0 (Varian Medical Systems, Palo Alto CA, USA).

## Results

### Original PTV and OAR’s volume and DVH data registered for each model

The mean ± SD of the PTV registered for each model were 91.4 ± 26.0 cm^3^, 99.5 ± 35.6 cm^3^, 82.0 ± 16.9 cm^3^, 136 ± 35.1 cm^3^, and 95.3 ± 24.5 cm^3^ for institutions A, B, C, D, and E, respectively. In the original OAR’s volume registered in each model, the mean ± SD of the rectal volume were 50.1 ± 13.7 cm^3^, 59.7 ± 24.9 cm^3^, 50.3 ± 14.2 cm^3^, 54.3 ± 21.9 cm^3^, and 45.2 ± 14.1 cm^3^ for institutions A, B, C, D, and E, respectively. The mean ± SD of the bladder volume were 151.3 ± 69.2 cm^3^, 165.1 ± 98.4 cm^3^, 179.5 ± 63.4 cm^3^, 80.7 ± 44.9 cm^3^, and 172.8 ± 101.7 cm^3^ for institutions A, B, C, D, and E, respectively. The mean ± SD of the rectal volume overlapping with PTV were 5.0 ± 1.4 cm^3^, 3.2 ± 1.4 cm^3^, 2.9 ± 1.2 cm^3^, 3.6 ± 1.3 cm^3^, and 4.6 ± 1.8 cm^3^ for institutions A, B, C, D, and E, respectively. The mean ± SD of the bladder volume overlapping with PTV were 8.3 ± 3.3 cm^3^, 12.5 ± 7.0 cm^3^, 8.9 ± 3.9 cm^3^, 5.9 ± 2.1 cm^3^, and 12.6 ± 4.4 cm^3^ for institutions A, B, C, D, and E, respectively. The mean ± SD of the V_overlap_/V_whole_ for the rectum were 10.4% ± 3.0%, 6.0% ± 2.7%, 5.8% ± 2.2%, 7.2% ± 2.8%, and 10.5% ± 3.8% for institutions A, B, C, D, and E, respectively. The mean ± SD of the V_overlap_/V_whole_ for the bladder were 6.4% ± 3.1%, 8.7% ± 3.9%, 5.1% ± 2.3%, 9.2 ± 10.2%, and 9.0% ± 4.4% for institutions A, B, C, D, and E, respectively.

**Fig. 1:**
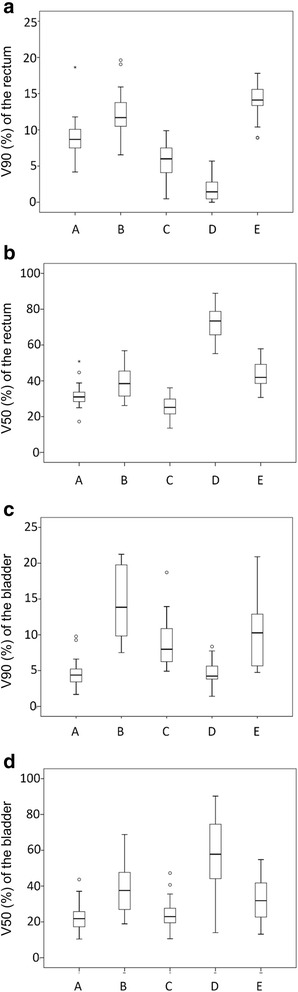
Box plots for rectal and bladder doses registered for each model at each institute. (**a**) The rectal volume receiving 90% of the dose (V90) in original dose volume histogram (DVH) curves for each model. (**b**) The rectal volume receiving 50% of the dose (V50) in original DVH curves for each model. (**c**) V90 of the bladder in original DVH curves for each model. (**d**) V50 of the bladder in original DVH curves for each model

In the original DVH data registered for each model, the mean ± SD of the rectal volume ratio receiving 90% of the prescribed dose (V90) were 11.2% ± 3.2%, 11.3% ± 3.3%, 5.7% ± 2.5%, 1.4% ± 1.3%, and 15.4% ± 3.6% at institutions A, B, C, D, and E, respectively. The mean ± SD of the rectal volume ratio receiving 50% of the prescribed dose (V50) were 37.5% ± 7.0%, 37.8% ± 9.1%, 25.2% ± 6.4%, 69.2% ± 12.6%, and 45.3% ± 6.5% at institutions A, B, C, D, and E, respectively. The mean ± SD of V90 of the bladder were 6.4% ± 3.1%, 16.4% ± 6.0%, 9.1% ± 3.6%, 5.8% ± 4.0%, and 10.9% ± 5.0% at institutions A, B, C, D, and E, respectively. The mean ± SD of V50 of the bladder were 27.9% ± 11.4%, 42.0% ± 14.9%, 24.5% ± 8.7%, 62.6% ± 20.1%, and 34.3% ± 14.3% at institutions A, B, C, D, and E, respectively. The box plots of the rectal and bladder dose are shown in Fig. 1. The median of each rectal dose for institute E were the highest among the sites. Those for institute C were the smallest. The median of each bladder dose for institute B was the highest.

### Manual optimization planning vs. KBP

**Fig. 2:**
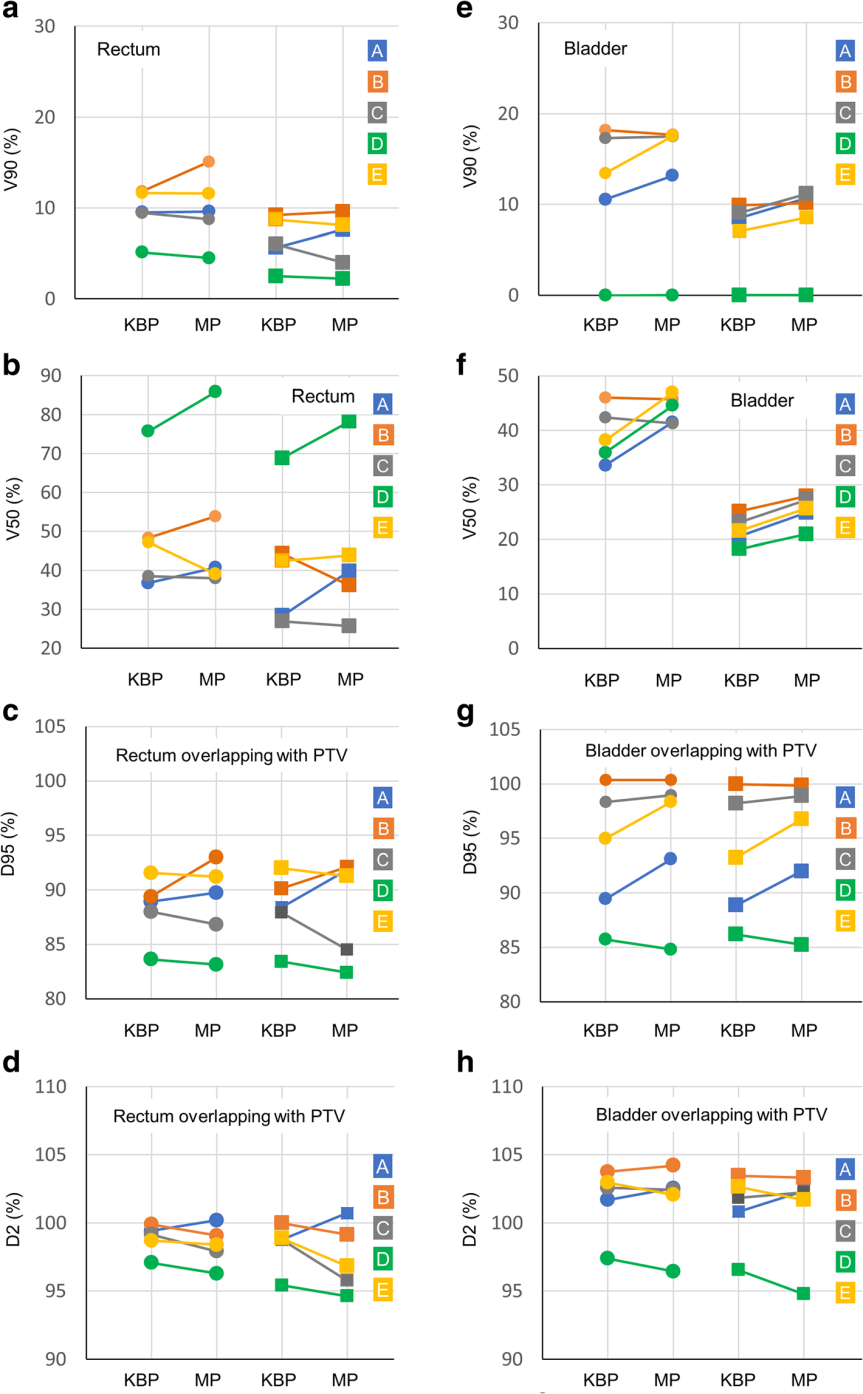
Comparison between knowledge-based planning (KBP) and manual optimization planning in rectal and bladder doses. (**a**) and (**e**) are the volume receiving 90% of the dose (V90) of rectum and bladder. (**b**) and (**f**) are the volume receiving 50% of the dose (V50) of rectum and bladder. (**c**) and (**g**) are the dose received by at least 95% of the volume (D95) of the rectum and bladder, and (**d**) and (**h**) are the dose received by at least 2% of the volume (D2) for the rectum and bladder. Circles in each graph are dosimetric parameters of case I and squares are case II. The colors of the circles, squares, and lines represent institutes. (Blue: **a**, Orange: **b**, Gray: **c**, Green: **d**, and Yellow: **e**)

In the rectal and bladder doses calculated by KBP and manual optimization planning, V90 and V50 are shown in Fig. 2 (a), (b), (e), and (f). In the V90 of the rectum, the mean ± SD of difference between KBP and manual optimization planning was 0.4% ± 1.6% and − 0.1% ± 1.5% in cases I and II, respectively. A negative value implies that dosimetric values for KBP were higher than those for manual optimization planning. In the V50 of the rectum, the mean ± SD of difference between KBP and manual optimization planning was 2.2% ± 6.9% and 2.6% ± 8.0% in cases I and II, respectively. For the V90 of the bladder, the mean ± SD of difference between KBP and manual optimization planning was 1.3% ± 2.0% and 1.0% ± 0.9% in cases I and II, respectively. For the V50 of the bladder, the mean ± SD of differences between KBP and manual optimization planning was 4.8% ± 5.0% and 3.6% ± 0.9% in cases I and II, respectively.

The dose received by at least 95% of the volume (D95) for the OARs is shown in Fig. 2 (c) and (g). For the D95 of the rectum, the mean ± SD of differences between KBP and manual optimization planning was 0.5% ± 1.9% and 0.1% ± 2.7% in cases I and II. For the D95 of the bladder, the mean ± SD of differences between KBP and manual optimization planning was 1.4% ± 2.0% and 1.2% ± 2.0% in cases I and II. There were no significant differences in each dosimetric parameter between the cases.

The dose received by at least 2% of the volume (D2) for the organs is shown in Fig. 2 (d) and (h). In the D2 for the rectum, the mean ± SD of the difference between KBP and manual optimization planning were − 0.5% ± 0.8% and − 0.9% ± 1.8% in cases I and II. In the D2 for the bladder, the mean ± SD of difference between KBP and manual optimization planning were − 0.1% ± 0.8% and − 0.2% ± 1.3% in cases I and II. There were no significant differences in each dosimetric parameter between the cases.

Various dosimetric values were calculated by KBP among institutes even if they used the same dosimetric parameters. Among institutions, the maximum differences in V90 for the rectum were 6.7% and 6.7%, V50 for the rectum were 39.0% and 41.9%, V90 of the bladder were 18.2% and 9.9%, and V50 of the bladder were 12.5% and 6.7% in cases I and II, respectively. These results suggested that each institutional KBP was useful in that particular institute regardless of the number of registered plans in the model, but the performance varied widely among the institutes.

### The relationship estimated dose and overlap volumes

**Fig. 3:**
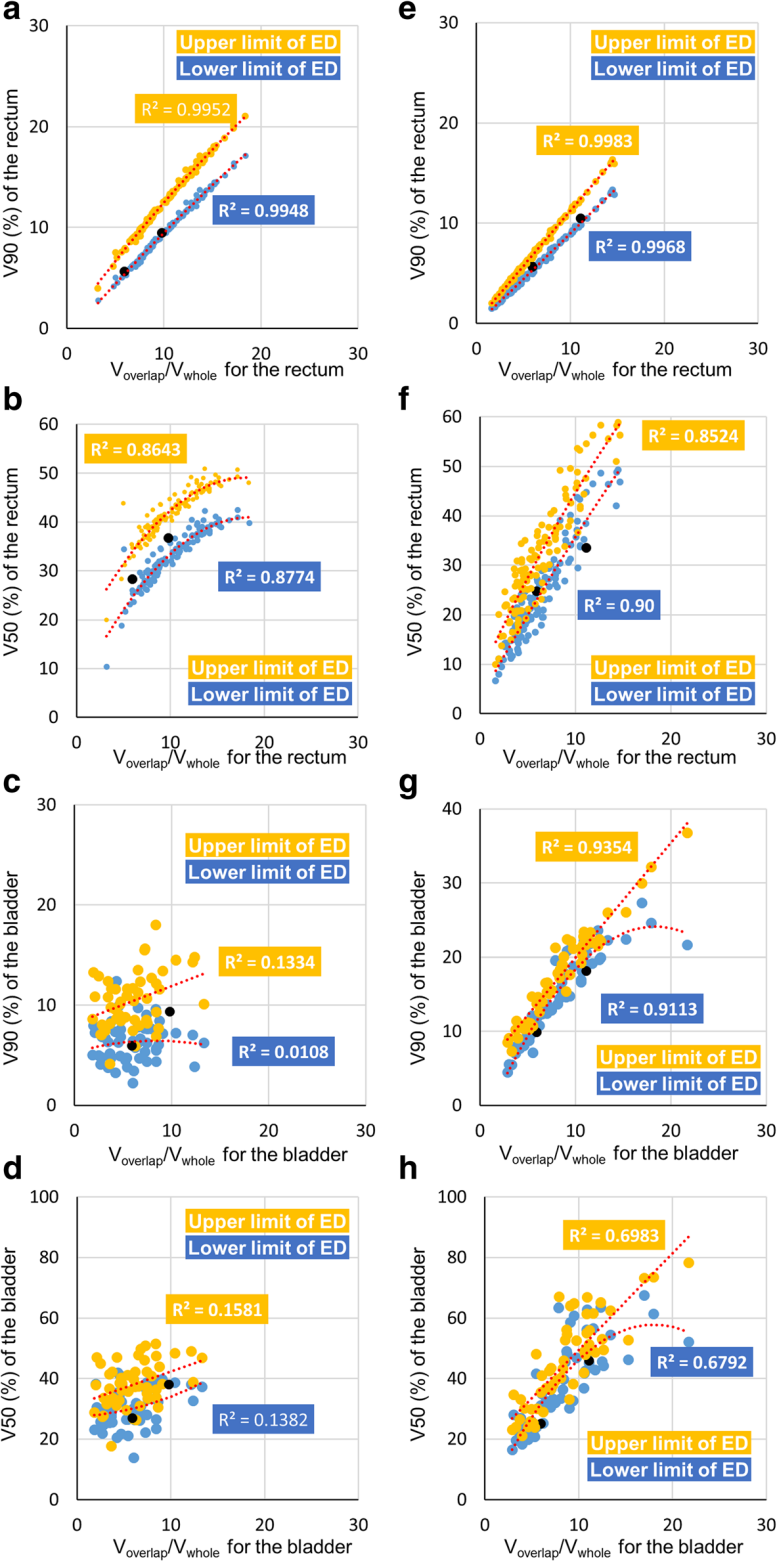
Relationship between estimated dose (ED) and ratio of an OAR’s volume overlapping with PTV to the whole organ volume (V_overlap_/V_whole_) for the rectum and bladder in institute **a** (**a**), (**b**), (**e**), (**f**) and **b** (**c**), (**d**), (**g**), (**h**). The horizontal axis is the V_overlap_/V_whole_ for the rectum (**a**, **b**, **c**, **d**) and V_overlap_/V_whole_ for the bladder (**e**, **f**, **g**, **h**). The vertical axis is the V90 to rectum (**a**, **c**) or bladder (**e**, **g**). The vertical axis is the V50 for the rectum (**b**, **d**) or bladder (**f**, **h**). Yellow dots represent the upper limit of ED and blue dots, the lower limit of ED. Red dotted lines with coefficients of determination (R^2^) are quadratic regression curves between each organ dose and V_overlap_/V_whole_ for organs. Black dots are calculated doses with knowledge-based planning (KBP) in cases I and II

Figure 3 shows the relationships between the upper limit and lower limit of ED and V_overlap_/V_whole_ for the rectum and the bladder, in institutes A and B. Dotted lines are quadratic regression curves between EDs and the V_overlap_/V_whole_. The black dots are dosimetric values calculated by KBP in cases I and II. Black dots were compiled with regression curves for each organ. The dosimetric values that were calculated by KBP were between curves of the upper and lower limits of ED or slightly lower than the curve of the lower limit of ED. In each organ, coefficients of determination (R^2^) of each dosimetric value and V_overlap_/V_whole_ for the rectum and the bladder are shown in Table 4. The R^2^ values of V90 were greater than those for V50, except at institution B for the rectum. In the bladder, the R^2^ of V90 were more than those for V50 at all institutions.

**Table 4:** Coefficients of determination (R^2^) of between each dosimetric value (V90 and V50) and ratio of an OAR’s volume overlapping with PTV to the whole organ volume (V_overlap_/V_whole_).vb

Institutions	Organs	Dosimetric Parameters	Coefficients of determination (R^2^) in the quadratic regression curves		
			DVH for registered plans	Upper limit of ED	Lower limit of ED
A	Rectum	V90	0.601	0.996	0.994
		V50	0.488	0.864	0.877
	Bladder	V90	0.865	0.998	0.997
		V50	0.781	0.852	0.903
B	Rectum	V90	0.254	0.133	0.011
		V50	0.293	0.158	0.138
	Bladder	V90	0.845	0.935	0.911
		V50	0.695	0.679	0.698
C	Rectum	V90	0.666	0.967	0.961
		V50	0.592	0.826	0.839
	Bladder	V90	0.885	0.931	0.962
		V50	0.620	0.594	0.649
D	Rectum	V90	0.529	0.997	0.995
		V50	0.485	0.494	0.519
	Bladder	V90	0.987	0.999	0.999
		V50	0.515	0.544	0.578
E	Rectum	V90	0.626	0.944	0.946
		V50	0.169	0.334	0.435
	Bladder	V90	0.971	0.994	0.994
		V50	0.804	0.849	0.866

*Abbreviations: DVH* dose volume histogram, *ED* estimated dose, *V90* the volume ratio of receiving 90% of the prescribed dose, *V50* the volume ratio of receiving 50% of the prescribed dose

### Estimated vs. calculated dose

**Fig. 4:**
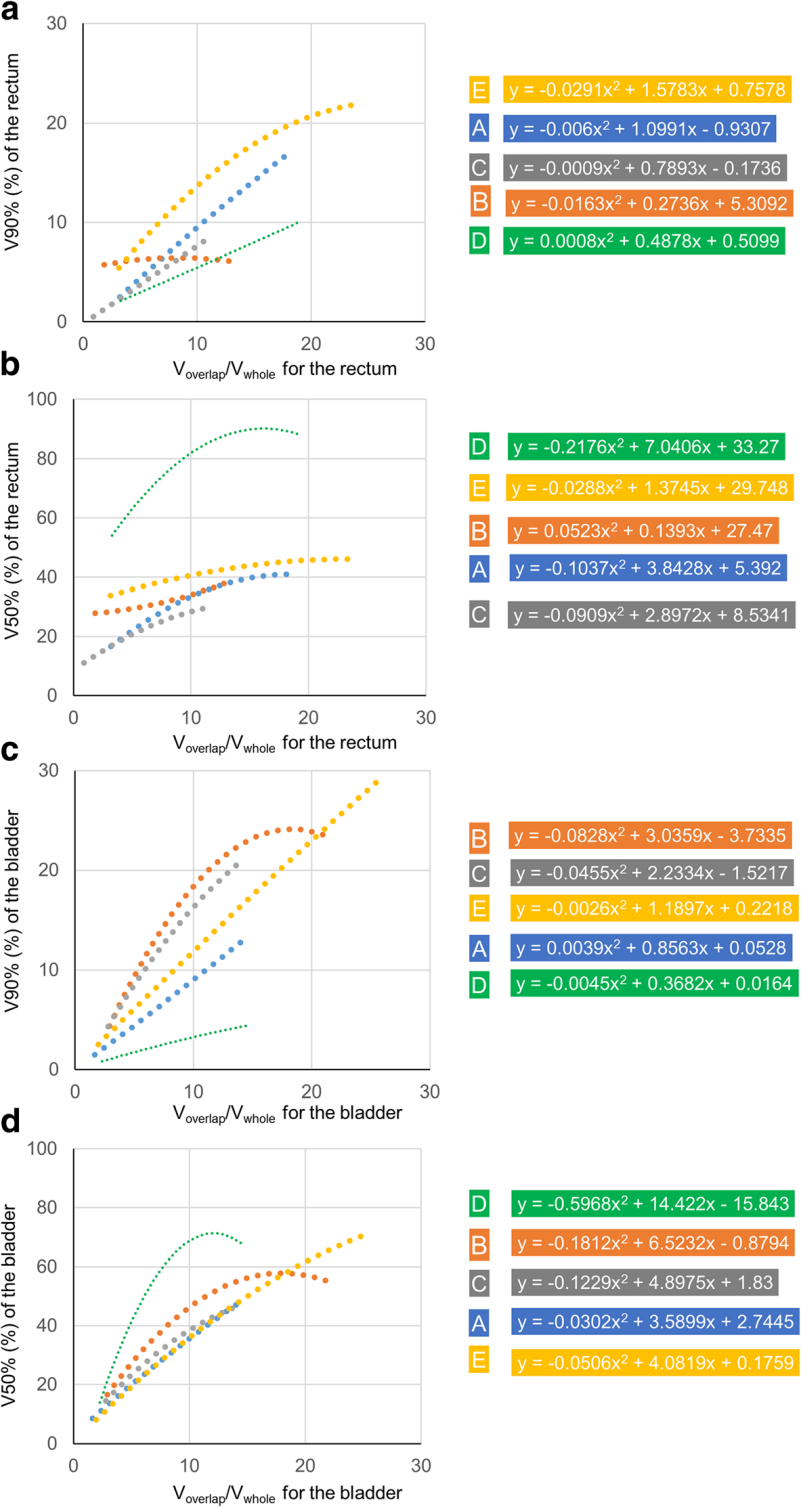
Quadratic regressions curves between lower limit of estimate dose (ED) and ratio of an OAR’s volume overlapping with PTV to the whole organ volume (V_overlap_/V_whole_) for the rectum and bladder with the formulas for all institutes. The horizontal axis is the V_overlap_/V_whole_ for the rectum (**a**, **b**) and bladder (**c**, **d**). The vertical axis is the volume receiving 90% of the prescribed dose (V90) to the rectum (**a**) and bladder (**c**). The vertical axis is the volume receiving 50% of the prescribed dose (V50) for the rectum (**b**) and bladder (**d**). The colors of the dotted lines represent institutes. (Blue: **a**, Orange: **b**, Gray: **c**, Green: **d**, and Yellow: **e**)

Quadratic regression curves between lower limit of ED and V_overlap_/V_whole_ for the rectum with the formulas for all institutes are shown in Fig. 4 (a), (b). In the V90 of the rectum (Fig. 4 [a]), four institutes except institute B had regression curves that tended to increase with increasing V_overlap_/V_whole_ for the rectum. In institute B, the regression curve was almost horizontal. The V90 dose in institute E was the highest of all V_overlap_/V_whole_ for the rectum. When V_overlap_/V_whole_ for the rectum was about 10%, the difference in the lower limits of ED between institutions D and E was > 8%. In the V50 for the rectum (Fig. 4 [b]), Institute D had the highest lower limit of ED in all V_overlap_/V_whole_ for the rectum. When the V_overlap_/V_whole_ for the rectum was 10%, the difference in lower limit of ED between institutes C and D was > 50%.

In the V90 and V50 for the bladder (Fig. 4 [c], [d]), the lower limit of ED curves for all institutes tended to show increases with increasing V_overlap_/V_whole_ for the bladder. In the V90 for the bladder (Fig. 4 [c]), when V_overlap_/V_whole_ for the bladder was 10%, the slopes of lower limits of ED for institutes B and C were steeper than those for institutions A, D, and E. In the V50 for the bladder (Fig. 4 [d]), V_overlap_/V_whole_ for the bladder was approximately 10%, the lower limits of ED were almost the same for institutes A, C, and E. The slope of the curves varied according to facilities. In among institutions, the maximum differences for lower limit of ED of V90 for the rectum were 8.2% and 5.7%, V50 for the rectum 53.5% and 45.0%, V90 for the bladder 15.1% and 9.4%, V50 to the bladder 33.1% and 26.0% when overlap volume with PTV was 10.0% and 6.0%, respectively.

## Discussion

In this study, five institutes used KBP for two cases each and the performance of the KBP models was compared among institutions. Some reports have evaluated the utility of KBP with one model [9–12]. This study uncovered that KBP performed effectively in five institutes, creating plans for clinical use. Each institute had its own plan design. KBP performed correctly regardless of the plan designs. This result suggests that the KBP models produced similar dose distributions as those of the model’s institutions with KBP. Additionally, in registered numbers of plans in each model, only 20 cases might be enough if there are large variations in the registered cases.

Kubo et al. [12] described that the dose coverage to the PTV was slightly inferior in KBP plans compared with manual optimization planning, as can be seen in values for D95 and D2. They used predicted priority values for PTV to confirm KBP predicted accuracy; these values might be underestimated to achieve the dose constraint objectives. In this study, the dose to the PTV was slightly inferior in KBP plans compared with manual optimization planning in some institutes, although there was no significant difference in D95 for the rectal and bladder volumes within the PTV. The first priority was reducing OAR dose for the KBP.

Schubert et al. have proven that it is possible to share models among different institutes in a cooperative framework [13]. Institutes in the report had the same plan design. In this study, in the KBP for multiple institutions, the maximum dosimetric differences for the V90 and V50 calculated with KBP among institutions were > 6.0% in cases I and II in both bladder and rectum. These results suggest that values calculated with KBP were influenced by plans registered in the model. Therefore, it depends on plan designs were matching between institutions whether the models made in other institutions can be shared.

Moore et al. found that that an OAR’s mean dose strongly correlated with the rectal and bladder volumes within the PTV [5]. In inverse planning, the understanding of geometric displacements of PTV and OARs led to predicting OAR dose and reducing the planner’s variations [5–7]. In this study, it was suggested that V90 and V50 had also strong correlation with the rectal and bladder volumes within the PTV in almost institutes. It was found that the correlation tendencies were different among institutes. To optimize the model for a case, it was acceptable to verify the relationship between OARs dose and the rectal and bladder volumes within the PTV.

Tol et al. [7] found that there were strong linear correlations (R^2^ = 0.94–0.99) between estimated and achieved mean doses in KBP. They derived the estimated mean dose from KBP models. The ED of the model was important for understanding the performance of KBP. In this study, EDs for V50 and V90 were compared between institutes. To reduce the volume, such as V50 and V90 for the OARs, leads to prevent radiation toxicity for the rectum and bladder. Peeters et al. argued that both intermediate and high doses to the anorectal wall volume should be considered to evaluate the risk of late GI toxicity [14]. Harsolia et al. found the volume of the bladder wall receiving ≥30 and ≥ 80 Gy predicted grade ≥ 2 late toxicity and grade 3 late toxicity [15]. In this study, it was indicated that the calculated OAR dose with KBP depended on registered plans in the model and correlated with OARs volumes in the PTV strongly. Thus, predicting OAR dose from the V_overlap_/V_whole_ for the rectum and bladder will be required to select the optimal model among several models.

In the relationships between OAR dose and the rectal and bladder volumes within the PTV, R^2^ values of V90 were higher than those of V50, except the rectum in institute B, because the OAR volume in the PTV affects the high dose region in the DVH curve [8]. In institute B, R^2^ values of the rectum were lower than those of other institutes. V90 for the rectum registered in the model was weak correlation with V_overlap_/V_whole_ although there were strong correlations in other institutions. In plan designs at institutions except institute B, V90 for the rectum depended on the rectal volume within the PTV. The correlation values between the R^2^ for EDs and dose for original DVH in the model were strong, 0.793 and 0.783 as Table 4. This result was showed the plan designs of plans registered in the models affected the relationships between ED and V_overlap_/V_whole_.

## Conclusions

It has been suggested that KBP performs correctly regardless of institutional plan design. KBP was able to reproduce dose distributions based on the experience of institutions. There was very wide variation in the organ dose calculated with KBP among sites. To share models for KBP, it will be necessary to determine whether the registered DVH curves in the models match the plan design. The models for the KBP were characterized with the ratio of OAR’s volume overlapping with the PTV to the whole organ volume.

## Abbreviations

CTV: Clinical target volume
D2: Dose received by at least 2% of the volume
D95: Dose received by at least 95% of the volume
DVH: Dose volume histogram
ED: Estimated doses
gEUD: Generalized equivalent uniform dose
GTV: Gross tumor volume
IMRT: Intensity-modulated radiotherapy
KBP: Knowledge-based planning
OAR: Organ at risk
PTV: Planning target volume
TPS: Treatment planning system
V50: Volume receiving 50% of the prescribed dose
V90: Volume receiving 90% of the prescribed dose
VMAT: Volumetric-modulated arc therapy
V_overlap_/V_whole_: Ratio of an OAR’s volume overlapping with PTV to the whole organ volume

## References

[1] Williams MJ, Bailey MJ, Forstner D, Metcalfe PE (2007). Multicentre quality assurance of intensity-modulated radiation therapy plans: a precursor to clinical trials. Australas Radiol.

[2] Chung HT, Lee B, Park E, Lu JJ, Xia P (2008). Can all centers plan intensity-modulated radiotherapy (IMRT) effectively? An external audit of dosimetric comparisons between three-dimensional conformal radiotherapy and IMRT for adjuvant chemoradiation for gastric cancer. Int J Radiat Oncol Biol Phys.

[3] Bohsung J, Gillis S, Arrans R, Bakai A, De Wagter C, Knöös T, Mijnheer BJ (2005). IMRT treatment planning—a comparative inter-system and inter-Centre planning exercise of the QUASIMODO group. Radiother Oncol.

[4] Wuthrick EJ, Zhang Q, Machtay M, Rosenthal DI, Nguyen-Tan PF, Fortin A (2015). Institutional clinical trial accrual volume and survival of patients with head and neck cancer. J Clin Oncol.

[5] Moore KL, Schmidt R, Moiseenko V, Olsen LA, Tan J, Xiao Y (2015). Quantifying unnecessary normal tissue complication risks due to suboptimal planning: a secondary study of RTOG 0126. Int J Radiat Oncol Biol Phys.

[6] Wu B, McNutt T, Zahurak M, Simari P, Pang D, Taylor R (2012). Fully automated simultaneous integrated boosted-intensity modulated radiation therapy treatment planning is feasible for head-and-neck cancer: a prospective clinical study. Int J Radiat Oncol Biol Phys.

[7] Tol JP, Dahele M, Delaney AR, Slotman BJ, Verbakel WF (2015). Can knowledge-based DVH predictions be used for automated, individualized quality assurance of radiotherapy treatment plans?. Radiat Oncol.

[8] Yuan L, Ge Y, Lee WR, Yin FF, Kirkpatrick JP, Wu QJ (2012). Quantitative analysis of the factors which affect the interpatient organ-at-risk dose sparing variation in IMRT plans. Med Phys.

[9] Tol JP, Delaney AR, Dahele M, Slotman BJ, Verbakel WF (2015). Evaluation of a knowledge-based planning solution for head and neck cancer. Int J Radiat Oncol Biol Phys.

[10] Fogliata A, Nicolini G, Clivio A, Vanetti E, Laksar S, Tozzi A (2015). A broad scope knowledge based model for optimization of VMAT in esophageal cancer: validation and assessment of plan quality among different treatment centers. Radiat Oncol.

[11] Wu H, Jiang F, Yue H, Li S, Zhang Y (2016). A dosimetric evaluation of knowledgebased VMAT planning with simultaneous integrated boosting for rectal cancer patients. J Appl Clin Med Phys.

[12] Kubo K, Monzen H, Ishii K, Tamura M, Kawamorita R, Sumida I (2017). Dosimetric comparison of RapidPlan and manually optimized plans in volumetric modulated arc therapy for prostate cancer. Phys Med.

[13] Schubert C, Waletzko O, Weiss C, Voelzke D, Toperim S, Roeser A (2017). Intercenter validation of a knowledge based model for automated planning of volumetric modulated arc therapy for prostate cancer. The experience of the German RapidPlan consortium. PLoS One.

[14] Peeters ST, Lebesque JV, Heemsbergen WD, van Putten WL, Slot A, Dielwart MF (2006). Localized volume effects for late rectal and anal toxicity after radiotherapy for prostate cancer. Int J Radiat Oncol Biol Phys.

[15] Harsolia A, Vargas C, Yan D, Brabbins D, Lockman D, Liang J (2007). Predictors for chronic urinary toxicity after the treatment of prostate cancer with adaptive three-dimensional conformal radiotherapy: dose-volume analysis of a phase II doseescalation study. Int J Radiat Oncol Biol Phys.

